# Low head circumference during early childhood and its predictors in a semi-urban settlement of Vellore, Southern India

**DOI:** 10.1186/s12887-019-1553-0

**Published:** 2019-06-06

**Authors:** Kulandaipalayam Natarajan Sindhu, Prashanth Ramamurthy, Karthikeyan Ramanujam, Ankita Henry, Joseph Dian Bondu, Sushil Mathew John, Sudhir Babji, Beena Koshy, Anuradha Bose, Gagandeep Kang, Venkata Raghava Mohan

**Affiliations:** 1 Division of Gastrointestinal Sciences, Christian Medical College, Vellore, Tamil Nadu India; 2 Rural Unit for Health and Social Affairs, Christian Medical College, Vellore, Tamil Nadu 632 209 India; 3 Department of Clinical Biochemistry, Christian Medical College, Vellore, Tamil Nadu India; 4 Low Cost Effective Care Unit, Christian Medical College, Vellore, Tamil Nadu India; 5 Developmental Pediatric Unit, Christian Medical College, Vellore, Tamil Nadu India; 6 Department of Community Health, Christian Medical College, Vellore, Tamil Nadu India

**Keywords:** Head circumference measurement, Maternal head circumference, Paternal head circumference, Growth, Nutrition, India

## Abstract

**Background:**

Stunting in developing countries continues to be a major public health problem. Measuring head circumference (HC) during clinical anthropometric assessment can help predict stunting. The aim of this study was to assess burden and determine the predictors of low HC (<− 2 SD) at birth and during first 2 years of life in a semi- urban settlement of Vellore.

**Methods:**

The study uses baseline data and serial HC measurements from the birth cohort of MAL-ED study, where 228 children from Vellore completed follow-up between March 2010 to February 2014. Analysis of baseline, maternal and paternal characteristics, micro-nutrient status and cognition with HC measurements was performed using STATA version 13.0 software.

**Results:**

The mean HC (±SD) at 1st, 12th and 24th month were 33.37 (1.29) cm, 42.76 (1.23) cm and 44.9 (1.22) cm respectively. A third of the infants (75/228) had HC less than − 2 SD at first month of life, and on follow-up, 50% of the cohort had HC ≤ -2 SD both at 12th and 24th month. Low HC measurements at all three time-points were observed for 21.6% (46/222) infants. Low HC was significantly associated with stunting in 37.3% (OR = 10.8), 57.3% (OR = 3.1) and 44.4% (OR = 2.6) children at 1st, 12th and 24th month respectively. Bivariate analysis of low HC (<− 2 SD) at 12th month showed a statistically significant association with lower socioeconomic status, low paternal and maternal HC and low maternal IQ. Multivariable logistic regression analysis showed maternal (AOR = 0.759, 95% CI = 0.604 to 0.954) and paternal (AOR = 0.734, 95% CI = 0.581 to 0.930) HC to be significantly associated with HC attained by the infant at the end of 12 months.

**Conclusions:**

One-third of the children in our cohort had low head circumference (HC) at birth, with one-fifth recording low HC at all time-points until 2 years of age. Low HC was significantly associated with stunting. Paternal and maternal HC predicted HC in children. HC measurement, often less used, can be a simple tool that can be additionally used by clinicians as well as parents/caregivers to monitor child growth.

**Electronic supplementary material:**

The online version of this article (10.1186/s12887-019-1553-0) contains supplementary material, which is available to authorized users.

## Background

Even though stunting among children from the developing world is on a decline over the last two decades, it still continues to be a major public health problem [1, 2]. The Global Nutrition report 2017 has estimated that 155 million children are stunted across 72 countries, with two of every five stunted children living in South Asia [2]. In India, nearly one third of under-five children are stunted according to the recent National Family Health Survey-4 (NFHS-4) [3]. Growth monitoring through periodic anthropometric measurements serves as an alarm for growth faltering in children, thereby signalling the need for appropriate and timely action, and this has been a routine practice incorporated within the health systems of many countries. The three commonly used parameters for monitoring growth in children include weight, length/height and head circumference (HC). However, measuring HC is not regularly done in many settings of developing countries, omitted even if done, with only weight and length/height measurements being predominantly taken in clinical anthropometric assessment and research studies [4].

Majority of the brain growth has been known to occur within the first two years of life and this steadily increases in volume up to adolescence. A low HC measurement can not only help predict and add on to the signs of stunting but can also predict brain development and cognition in children during their pre-school years [5]. Studies have shown that serial HC measurements during early childhood is a robust reflector of the brain volume and can help plot the trajectory of brain growth, thereby determining the cognitive functionality in later life [6–8]. A prospective study from Southern India has shown HC to be positively correlated with learning and visio-spatial ability in children aged 9 to 10 years [5]. Low HC measurements at birth and differential HC measurements in infants have also shown to be associated with social impairment, symptoms of autism spectrum disorders and motor delays later [9, 10].

Multiple factors influence HC in children through complex pathways, some of them being maternal education, maternal intelligent quotient, maternal body-mass-index, socio-economic profile, birth weight, exclusive breast feeding, maternal smoking and others [11–17]. The height, weight and HC of parents, especially the maternal HC, have also shown to significantly influence the HC of infants suggesting a strong intra-uterine and genetic influence [18–20]. Anaemia, low zinc and Vitamin A levels have been observed in children with stunting from cross-sectional studies and this is catalysed by the presence of concomitant inflammation as indicated by the detection of acute phase reactants such as α-1-acid glycoprotein [21, 22]. However, the association of micronutrients specifically with HC in children has not been studied in the developing world where micronutrient deficiency among children is widely prevalent. A recent study from rural Bangladesh has highlighted that either WASH or nutrition imparted as an intervention independently, had an improvement on the HC Z-scores in children of the intervention arm when compared to their age-matched controls. However, no difference was seen when combined Water, sanitation and Hygiene (WASH) along with nutrition as a package was given to the intervention arm when compared to control arm. This implicates the influence of a complex array of factors on HC and anthropometry on the whole, direct and indirect, along with the inherent maternal and paternal influences [23].

This study aims to assess the burden of low HC (<− 2 SD) at birth and its progress during first 2 years of life among children residing in a semi-urban settlement of Vellore. The study also determined the effect of socio-demographic, parental characteristics and micronutrient status on HC of children during the first 2years of life.

## Methods

### Study design

The present study uses baseline data and serial HC measurements from the birth cohort of MAL-ED (The Aetiology, Risk Factors, and Interactions of Enteric Infections and Malnutrition and the Consequences for Child Health) study, a multi-country birth cohort study, which was established at eight sites and was led by the Fogarty International Centre of the National Institutes of Health and the Foundation for the National Institutes of Health [24]. The aim of the MAL-ED study was to study the multiple effects and impact of enteric infections and malnutrition on child growth, cognition, and response to early childhood vaccination.

### Setting

Vellore town (12.9° N, 79.1° E), situated about 137 km from the city of Chennai (capital of the state of Tamil Nadu in south India), was one of the eight sites of MAL-ED study. The study site established at Vellore is a semi-urban settlement that comprises of a stretch of densely populated eight neighbourhoods with a total population of around 13,000. This section of the predominantly urban poor is catered to for its health needs by the government UPHC (Urban Primary Heath Centre) and the LCECU (Low Cost Effective Care Unit), which is a part of the community out-reach programme of the Christian Medical College, Vellore. LCECU has been closely working in this area over the last few decades to improve health, and has been serving this community, enabling referrals to the hospital where necessary. The site established a birth cohort of infants who were born healthy and were recruited within a window period of 17 days following birth.

### Study period

The study was carried over a period of 4 years from March 2010 and ended in February 2014. Enrolment was completed in February 2012 and the last child completed the 24-month follow-up in February 2014.

### Study participants

The inclusion criteria were the child being born as a singleton, parent/primary caregiver of the child being a permanent resident of the study area and those willing to permit home visits by the designated field staff. Parents/primary caregivers of the child who were likely to be away from the study site for more than 30 days during the study, new-borns of teenage mothers, prolonged hospitalization of the neonate at birth, diagnosed with a chronic condition or enteropathy and those who weighed less than 1500 g at the time of enrolment were excluded from the study. An informed written consent was obtained from the parent/primary caregiver of the child after having explained to him/her the purpose of the study in the local language - Tamil, a Dravidian language spoken in Vellore and the rest of the state of Tamil Nadu.

The new-born infants were enrolled in the study and followed up between March 2010 to February 2014. Field workers who were residents of the same community were selected for the cohort follow-up, and this strengthened the establishment of a smooth and robust rapport with the families. The infants were followed up at home by the designated field worker at specific time points as per protocol. Sick infants or those needing physician care were referred to a study clinic established in the study area, and further to LCECU if needed.

### Sample size calculation

A prior population survey was performed in the study area before the commencement of enrolment. Using the number of women in the reproductive age enumerated in the survey, it was estimated that approximately 200 infants would be born within the MAL-ED study area in the enrolment period of 2 years. This led to the enrolment of approximately 10 infants every month over a period of 2 years [24, 25].

### Study procedures and measurements

**Fig. 1:**
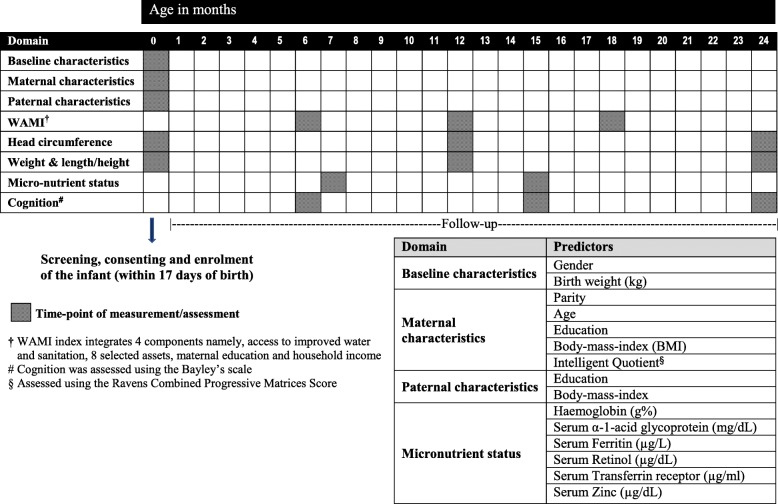
Schematic representation of the study flow with follow-up time-points of recording baseline, paternal and maternal characteristics, weight, length/height, head circumference and assessment of micronutrient status

Following enrolment in the study, date of birth, gender and birth weight of the children were recorded. A structured questionnaire was used to collect socio-demographic and parental characteristics that included paternal and maternal age, education and socio-economic status (SES). Parental body-mass-index (BMI), HC and maternal intelligence quotient (IQ) were measured and documented as per protocol (Fig. 1). Maternal age was grouped as young mother (≤23 years) and older mother (> 23 years) using the median cut-off value. Parental education was categorised as uneducated, primary (1st - 5th grade), secondary (6th - 10th grade) and high school (>11th grade). SES was measured using the WAMI index (access to improved Water and sanitation, eight selected Assets, Maternal education and household Income), developed to measure SES across diverse settings of low-and middle-income countries [26]. The score further led to the stratification of SES into low, middle and high using tertiles of the overall score. Maternal and paternal body-mass-indices were categorized as underweight (BMI < 18.5), normal (BMI 18.5–24.9) and overweight (BMI ≥25).

Birth weight was classified as normal, low and very low were if the birth weights were >  2.5 kg, 2–2.49 kg and < 1.99 kg respectively. Anthropometry including head circumference (HC) (occipito-frontal diameter) of the child were measured at 3 time points: at recruitment, 12th month and 24th month as shown in Fig. 1. The measurements were performed and recorded by a trained study nurse and was measured to the nearest 0.1 cm by a non-expandable HC measuring band made of synthetic Teflon material. HC in children was classified using the WHO head circumference-for-age Z-scores [27]. Low HC in these children was defined as a measurement less than − 2 SD. Wasting in the child was defined as weight-for-height (W/H) below − 2 SD and stunting as height-for-age (H/A) below − 2 SD using the WHO Child Growth Standards median [28]. Paternal and maternal HC measurements were categorized as low and normal using the median cut-off due to lack of standard HC reference charts for Indian adult population [29]. Maternal intelligence was assessed by the study psychologist using the Ravens Combined Matrices Score (RCM) [30]. The RCM scores classified the mothers as those with low IQ who scored less than or equal to 33rd centile and normal or high IQ with scores more than 33rd centile. Cognition in children was assessed using the Bayley’s scale at 6th, 15th and 24th month [31]. Micro-nutrient status of the children that incorporated levels of haemoglobin (g%), ferritin (ng/ml), retinol (g/L), transferrin receptor (mg/L) and zinc (μg/dL) were quantified by serology collected at 7th and 15th month of age. In conjunction, α-1-acid glycoprotein (mg/dL), was measured, the presence of which is a surrogate marker for active inflammation underlying sub-clinical infections and can lead to low levels of micronutrients in children [32]. The azide methaemoglobin method was employed for Haemoglobin estimation using a Hemocue (a battery driven photometer with disposable cuvettes) and anaemia was defined using the World Health Organisation’s definition of Haemoglobin less than 11 g/dL [33]. Serum ferritin (male: 22–322 ng/ml; female: 10–290 ng/ml), transferrin receptor (1.9–5 mg/L), zinc (75–120 μg/dL) and α-1-acid glycoprotein (50–200 mg/dL) were classified as low and normal using standard references [34, 35]. Serum retinol was estimated using High Performance Liquid Chromatography (HPLC) and a level < 0.2 g/L was considered as low [36].

### Statistical analysis

Data were entered using a double-entry database application and stored at the Data Coordinating Center (DCC) of MAL-ED established at the Fogarty International Center [24]. All analyses were performed using Stata version 13 (StataCorp. 2013. Stata Statistical Software: Release 13. College Station, TX: StataCorp LP). Descriptive statistics were computed and presented as proportions along with *p*-values within each variable. HC, stunting (H/A) and wasting (W/H) were calculated as proportions less that − 2 SD. A bi-variate analysis was performed to investigate or identify relationships between HC and socio-demographic variables, parental characteristics and micronutrient levels in the infant using Chi-square test, and odds ratios (OR) as well as 95% confidence intervals (CI). A bivariate analysis was also performed to study association between HC and stunting measured at all three time-points, to further generate ORs. To adjust for confounders, the significant variables by bivariate analysis were modelled using a multivariable logistic regression analysis and the adjusted odds ratios (AORs) with 95% confidence intervals (CI) were estimated. All variables in the regression model were imputed as categorical variables except paternal and maternal HC which were used as continuous variables. *P*-values presented are two-sided and *p*-value < 0.05 was considered as statistically significant. We used Hosmer-Lemeshow goodness-of fit-test to assess the model fit. The test (Chi-square value = 3.20, *p* = 0.92), suggested that the model showed a good fit for the covariates used. Also, we measured the area under the curve (AUC) which showed a value of 0.7188 substantiating the model with a good fit.

## Results

A total of 301 pregnant women (in their third trimester) consented to participate in the study and were followed until delivery. Following delivery, 251 infants were enrolled in the study. The 50 infants who thereby did not participate in the study comprised of 10 infants whose mothers withdrew consent following delivery and 40 infants did not meet the inclusion criteria. Overall, 228 (90.9%) children completed the 24th month follow-up with 23 (9.1%) children accounting for lost-to-follow, of who 15 (65.2%) had migrated from the study area.

The baseline, paternal and maternal characteristics are presented in Table 1. Of the 228 children, there were 105 (46%) males and 123 (54%) females. A parity of more than two was documented for 91/226 (40%) mothers. The mean birth weight of the cohort was 2.89 kg (SD = 0.44) with 32/223 (14%) low birth weight and 5/223 (2%) very low birth weight infants. The mean age of the mothers at the time of enrolment was 23.9 (SD = 4.2) years. The average paternal and maternal years of schooling were 6.91 (SD = 3.81) and 6.38 (SD = 3.81) years respectively with 26/226 (11%) mothers and 30/212 (14%) fathers having had no formal schooling. The mean maternal body mass index (BMI) was 22.04 (SD = 3.95) kg/m^2^ with 46/226 (20%) mothers being underweight and 48/226 (21%) overweight. Similarly, mean paternal body mass index (BMI) was 23.01 (SD = 4.25) kg/m^2^ with 20/205 (10%) fathers being underweight and 54/205 (26%) overweight. The maternal IQ assessment showed that 81/228 (36%) mothers scored within the lower third of the tertile. There was no variation in the WAMI scores over the 3 time points of measurement with 69 (31%) infants falling within the lower tertile at the first assessment (6th month).

**Table 1:** Baseline, maternal and paternal characteristics of the study participants *(N = 228)*

Variable		Category	*n*	%	*p-*value
Gender *(n = 228)*		Male	105	46	0.23
		Female	123	54	
Birth weight (kg) *(n = 223)*		Very Low Birth weight (< 1.99 kg)	5	2	**< 0.001**
		Low birth weight (2–2.49 kg)	32	14	
		Normal birth weight (≥ 2.5 kg)	186	84	
Socio-economic status (WAMI^a^)	6th month *(n = 225)*	Low (≤ 33rd centile)	69	31	**< 0.001**
		Middle and High (> 33rd centile)	156	69	
	12th month *(n = 228)*	Low (≤ 33rd centile)	74	32	**< 0.001**
		Middle and High (> 33rd centile)	154	68	
	18th month *(n = 228)*	Low (≤ 33rd centile)	72	32	**< 0.001**
		Middle and High (> 33rd centile)	156	68	
Parity of the mother (*n = 226)*		> 2	91	40	**< 0.001**
		≤ 2	135	60	
Age of the mother *(n = 226)*		< 23 years	96	42	0.109
		≥ 23 years	130	58	
Mother’s education *(n = 226)*		No schooling	26	11	**< 0.001**
		Primary (1st to 5th grade)	54	24	
		Secondary (6th to 10th grade)	110	49	
		High school (>11th grade)	36	16	
Father’s education *(n = 212)*		No schooling	30	14	**< 0.001**
		Primary (1st to 5th grade)	56	26	
		Secondary (6th to 10th grade)	109	52	
		High school (>11th grade)	17	8	
Mother’s BMI *(n = 226)*		Under-weight (< 18.5)	46	20	**< 0.001**
		Normal (18.5–24.9)	132	59	
		Over weight (≥ 25)	48	21	
Father’s BMI *(n = 205)*		Under-weight (< 18.5)	20	10	**< 0.001**
		Normal (18.5–24.9)	131	64	
		Over weight (≥ 25)	54	26	
Mother’s IQ (RCM^b^) *(n = 228)*		Low (≤ 33rd centile)	81	36	**< 0.001**
		Normal and High (> 33rd centile)	147	64	

^a^Socio-economic index that integrates 4 components namely, access to improved water and sanitation, 8 selected assets, maternal education and household income

^b^Ravens Combined Matrices Score

data in bold represents *p*-value <0.05

**Fig. 2:**
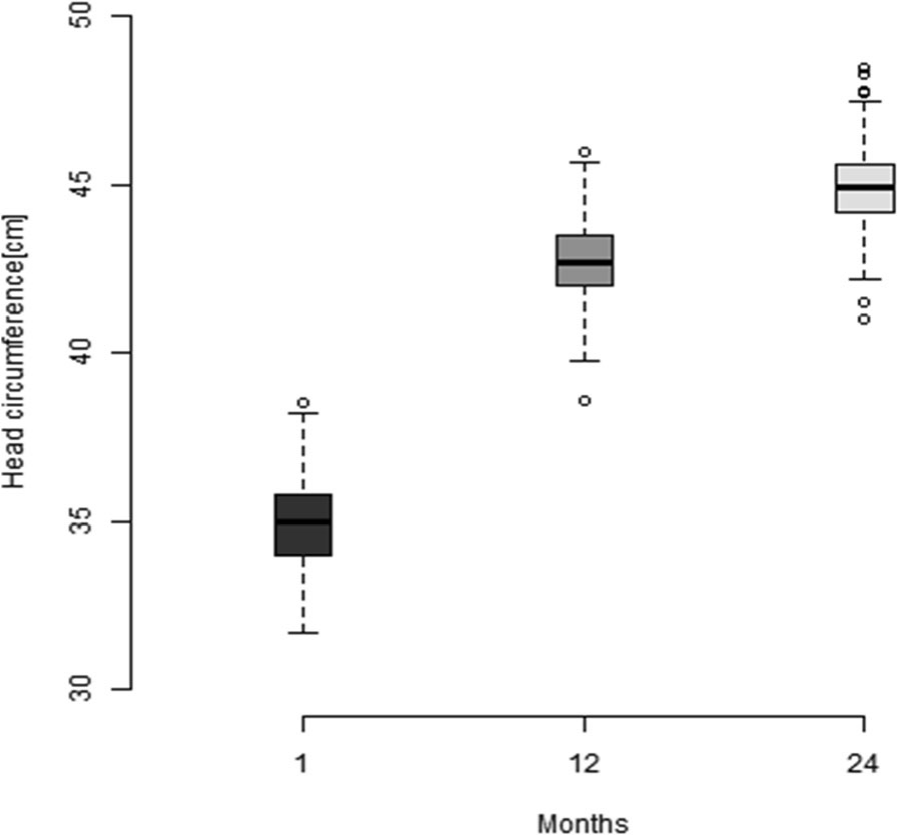
Box-and-whisker plot showing mean head circumference measurements of the children at 1st month (enrolment), 12th month and 24th month of age

The mean maternal and paternal HC (±SD) were 51.63 (1.57) cm and 53.3 (1.47) cm respectively. The mean HC (±SD) of the infants at recruitment (1st month), 12th month and 24th month were 33.37 (1.29) cm, 42.76 (1.23) cm and 44.9 (1.22) cm respectively (Table 2). About a third of the infants (75/228) had HC less than − 2 SD at first month of life. This was followed by about 51.8 and 51.5% of the cohort progressing to have HC < -2 SD measured at the 12th month and 24th month respectively (Table 2, Fig. 2). Among the children with a low HC at recruitment, 47.5% (56) were males and 52.5% (62) were females with no significant difference [*p*-value = 0.705, OR = 0.9 (0.51–1.570)]. Low HC measurements at all three time-points were observed for 21.6% (46/222) infants, with normal HC measurements being observed for 34.2% (76/222) children at all time-points of measurement in the cohort. Stunting was observed in 15.8, 31.4 and 44.5% of the cohort at 1st month, 12th month and 24th month respectively with 19.2, 15.5 and 11% being wasted at the same time-points. Low HC was observed in 37.3% [*p*-value < 0.001, OR = 10.8 (4.6–25.3)], 57.3% [*p*-value < 0.001, OR = 3.1(1.7–5.7)] and 44.4% [*p*-value < 0.001, OR = 2.6 (1.5–4.4)] of children with stunting at 1st month, 12th month and 24th month respectively. Similarly, low HC was seen in 36.6% [*p*-value < 0.001, OR = 4.6 (2.2–9.3)], 23.9% [*p*-value < 0.001, OR = 4.6 (1.9–11)] and 17.1% [*p*-value < 0.05, OR = 4.3 (1.6–12)] of children with wasting at 1st month, 12th month and 24th month respectively (Table 3).

**Table 2:** Head circumference (HC) measurements, stunting and wasting in the cohort at 1st, 12th and 24th month of age

Time point of measurement	Mean HC in cm (SD)	Mean HC Z-score (SD)	HC less than − 2 SD (%)	Stunting less than − 2 SD (%)	Wasting less than − 2 SD (%)
1st month *(n = 228)*	33.37 (1.29)	−1.50 (1.01)	75 (32.9)	36 (15.8)	43 (19.2)
12th month *(n = 226)*	42.76 (1.23)	−2.02 (0.80)	117 (51.8)	71 (31.4)	35 (15.5)
24th month *(n = 227)*	44.9 (1.22)	−2.00 (0.78)	117 (51.5)	101 (44.5)	25 (11)

**Table 3:** Bivariate analysis of head circumference (HC) with stunting and wasting at 1st, 12th and 24th month of age

Time point of measurement		Stunting (H/A)		*p-*value	OR (95% CI)	Wasting (W/H)		*p-*value	OR (95% CI)
		Stunting < − 2 SD (%)	No stunting (%)			Wasting < − 2 SD (%)	No wasting (%)		
1st month *(n = 228)*	Low HC	28 (37.3)	47 (62.7)	**< 0.001**	10.8 (4.6–25.3)	26 (36.6)	45 (63.4)	**< 0.001**	4.6 (2.2–9.3)
	Normal HC	8 (5.2)	145 (94.8)			17 (11.1)	136 (88.9)		
12th month *(n = 226)*	Low HC	50 (42.7)	67 (57.3)	**< 0.001**	3.1 (1.7–5.7)	28 (23.9)	89 (76.1)	**< 0.001**	4.6 (1.9–11)
	Normal HC	21 (19.3)	88 (80.7)			7 (6.4)	102 (93.6)		
24th month *(n = 227)*	Low HC	65 (55.6)	52 (44.4)	**< 0.001**	2.6 (1.5–4.4)	20 (17.1)	97 (82.9)	**< 0.05**	4.3 (1.6–12)
	Normal HC	74 (32.7)	36 (67.3)			5 (4.5)	105 (95.5)		

data in bold represents *p*-value <0.05

More than half of the children were anaemic at 7th and 15th month of age (Table 4). α-1-acid glycoprotein level was found to be elevated in 36 and 42% of the infants at 7th and 15th month respectively. Low serum ferritin was observed in a quarter of the children at the 7th month that significantly increased to 59% by the 15th month. Low serum retinol and Zinc levels were present in 19 and 51% of the infants respectively at the 7th month but at the 15th month, the number of children with low serum retinol declined to 13%. However, the number of children with low serum Zinc level significantly increased to 73%. An abnormal level of Transferrin receptor level was seen in 37% of the infants which remained unchanged when measured at the 15th month.

**Table 4:** Micronutrient status of the infants at 7th and 15th month of birth

Variable	Category	7th month			15th month			*p-*value
		*n*	Freq	%	*n*	Freq	%	
Haemoglobin (g%) *(n = 228)*	Anaemia	228	117	51.3	227	128	56.1	0.20
	Normal		111	48.7		99	44	
α-1-acid glycoprotein (mg/dL) *(n = 212)*	Elevated	212	76	35.8	225	94	42	0.33
	Normal		136	64.2		131	58	
Ferritin (ng/ml) *(n = 225)*	Low	225	52	23.1	221	130	59	**< 0.001**
	Normal		173	76.9		91	41	
Retinol (μg/dL) *(n = 204)*	Low	204	40	19.6	226	29	13	**< 0.01**
	Normal		164	80.4		197	87	
Transferrin receptor (mg/L) *(n = 214)*	Low	214	80	37.4	225	80	36	0.91
	Normal		134	62.6		145	64	
Serum Zinc (μg/dL) *(n = 217*	Low	217	110	50.7	226	165	73	**< 0.001**
	Normal		107	49.3		61	27	

data in bold represents *p*-value <0.05

Bivariate analysis of low HC at the end of 12th month (<− 2 SD) with the baseline characteristics and micronutrient status of the infants showed a significant association with low socioeconomic status, low paternal and maternal HC and a low maternal IQ (Table 5). Among the infants who had a HC < -2 SD at first month, 45.3% (34/75) showed poor cognition (< 33rd centile) measured at 6th month when compared to those with a normal HC, however this was not statistically significant (Chi-square = 0.002, *p*-value = 0.93). At 1 year of age, 32.4% (38/117) children with HC < -2 SD had poor cognition and there was no significant association with the 15th month cognition scores (Chi-square = 0.026, *p*-value = 0.87). No significant association was elicited between low HC and poor cognition (35/117) measured at 24th month (Chi-square = 0.567, *p*-value = 0.45).

**Table 5:** Bi-variate analysis of baseline, maternal and paternal characteristics; micro-nutrient status and cognition with HC measurement (12th month)

Predictor	Head circumference (cm)		Chi-squared value	*p*-value	OR (95% CI)
	<−2 SD (%)	Normal (%)			
Low birth weight (< 2.5 kg)	93 (50)	21 (58.3)	0.838	0.360	1.40 (0.64–3.10)
WAMI (6th month) (<33rd centile)	43 (37.1)	26 (24.1)	4.431	**0.035**	**1.86 (1.01–3.47)**
WAMI (12th month) (<33rd centile)	46 (39)	28 (25.7)	4.55	**0.033**	**1.85 (1.01–3.40)**
WAMI (24th month) (<33rd centile)	31 (26.5)	18 (16.7)	3.18	0.074	1.80 (0.89–3.68)
Low maternal HC (cm)	72 (62.6)	43 (42.2)	9.07	**0.003**	**2.29 (1.28–4.11)**
Low paternal HC (cm)	60 (56.1)	39 (39.8)	5.42	**0.02**	**1.93 (1.06–3.50)**
Parity of the mother (> 2)	68 (59.6)	65 (58.6)	0.02	0.87	0.96 (0.54–1.68)
Age of the mother (< 23 years)	48 (41.4)	47 (43.1)	0.07	0.80	0.93 (0.53–1.63)
Low maternal education	46 (39.7)	34 (31.2)	1.75	0.185	1.45 (0.80–2.61)
Low paternal education	48 (42.8)	38 (38.4)	0.43	0.509	1.20 (0.66–2.17)
Low maternal BMI	27 (23.1)	18 (16.7)	1.44	0.23	1.50 (0.73–3.10)
Low paternal BMI	13 (12.2)	7 (7.1)	1.45	0.23	1.79 (0.63–5.59)
Low maternal IQ (<33rd centile)	52 (44.1)	28 (25.7)	8.38	**0.004**	**2.28 (1.25–4.17)**
Exclusively breastfeeding (≤4 month)	109 (93.2)	97 (89)	1.42	0.49	1.68 (0.66–4.29)
Anaemia	62 (53)	55 (51)	0.09	0.75	1.08 (0.62–1.89)
Elevated α-1-acid glycoprotein (mg/dL)^a^	41 (37.9)	35 (33.9)	0.362	0.55	1.18 (0.65–2.17)
Low Ferritin (μg/L)^a^	31 (26.7)	21 (19.4)	1.66	0.19	1.51 (0.77–2.99)
Low Retinol (μg/dL)^a^	21 (20.2)	19 (19.2)	0.032	0.85	1.06 (0.50–2.26)
Low Transferrin receptor (μg/ml)^a^	66 (60.6)	67 (64.4)	0.34	0.56	0.84 (0.46–1.53)
Low Serum Zinc (μg/dL)^a^	61 (54.4)	48 (46.1)	1.49	0.22	1.39 (0.79–2.47)

^a^Measured at 7th month

data in bold represents *p*-value <0.05

**Fig. 3:**
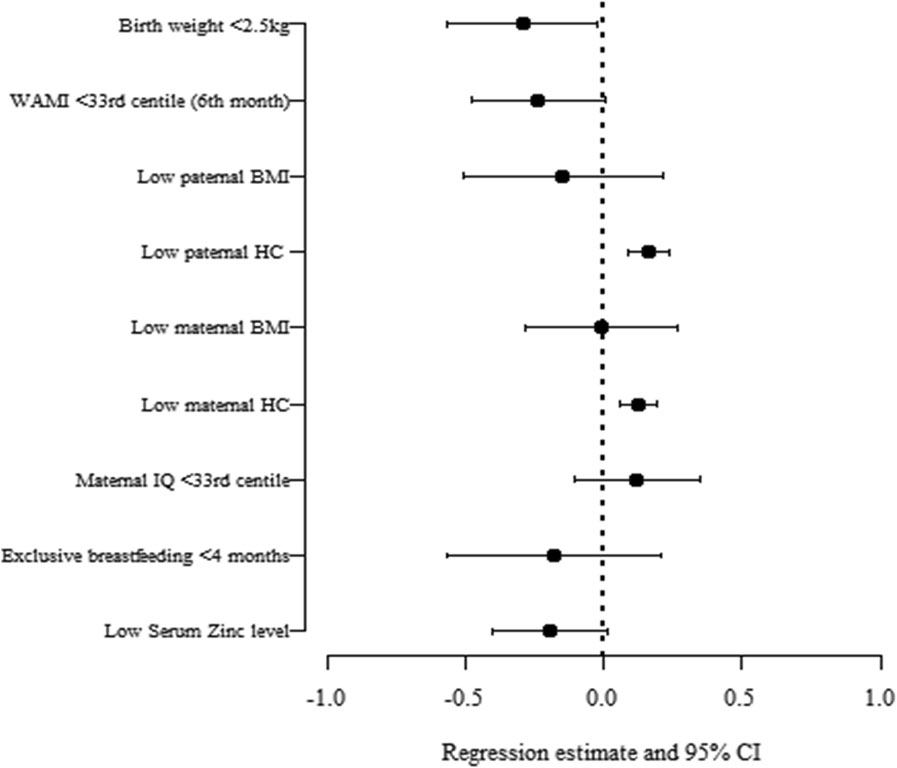
Multivariate regression analysis of the significant predictors of 12th month head circumference measurement

Multivariable logistic regression analysis for the significant predictors of low HC at 12th month (*n* = 190) is shown in Table 6 and represented in Fig. 3. Multivariable regression analysis showed that maternal and paternal HC were significantly associated with the HC attained by the infant at the end of 12th month. However, the above was not observed when the same predictors were compared with the HC measured at 24th month of age.

**Table 6:** Predictors of low head circumference (HC) at 12th month (n = 190) using multivariable logistic regression analysis

Predictor		β-coefficient (95%CI)	Adjusted OR (95% CI)	*p*-value
Birth weight	< 2.5 kg	0.491 (−0.344 to 1.325)	1.633 (0.709 to 3.764)	0.249
	≥2.5 kg	–	–	
WAMI (6th month)	<33rd centile	0.444 (−0.306 to 1.195)	1.560 (0.736 to 3.307)	0.246
	≥33rd centile	–	–	
Low paternal HC^a^		−0.308 (−0.544 to − 0.073)	0.734 (0.581 to 0.930)	**0.01**
Paternal BMI	Low	0.033 (−1.079 to 1.147)	1.034 (0.340 to 3.149)	0.952
	Normal	–	–	
Low maternal HC^a^		−0.275 (− 0.504 to − 0.046)	0.759 (0.604 to 0.954)	**0.018**
Maternal BMI	Low	0.110 (−0.727 to 0.947)	1.116 (0.483 to 2.579)	0.797
	Normal	–	–	
Maternal IQ	<33rd centile	−0.665 (−1.375 to 0.044)	0.514 (0.253 to 1.046)	0.066
	≥33rd centile	–	–	
Exclusive breast feeding	< 4 months	1.187 (−0.107 to 2.483	3.279 (0.898 to 11.977)	0.072
	≥4 months	–	–	
Serum Zinc level (μg/dL)	Low	0.441(−0.194 to 1.078)	1.556 (0.823 to 2.939)	0.173
	Normal	–	–	

^a^HC was used as a continuous variable for regression analysis

data in bold represents *p*-value <0.05

## Discussion

This is a prospective birth cohort study to estimate and examine factors influencing low HC in the first 2years of life in a semi-urban settlement of south India. This intensively followed-up cohort had a drop-out rate of less than 10% and no significant differences were observed between the baseline characteristics of children who were lost to follow-up or for those with missing data, with the cohort that completed the study follow-up at 24 months (Additional file 1: Table S7). We observed that there were no gender differences in HC measurements at 1 month of age and this was similar to the observation made by Veena et al. [5]. Our study showed one-third of the children started with a low HC at birth. This proportion in the cohort increased to 50% with low HC at the end of 1 year, and thereon persisted with no further change in the cohort at 2 years of life. Hence, this is a significant one-third of the population beginning very early in life with a reduced HC and is substantiated by the fact that Indian children start off right in-utero with a low HC [37]. The cohort had about a fifth of the children who recorded low HC at birth and continued to have low HC at all time-points until 2 years of age. This indicates that growth faltering that began early in-utero can continue to persist without catching up upto the first 2 years of life. This could further herald an array of effects of stunting that encompass linear growth failure in children such as repeated infections, poor cognition and further, chronic diseases in adulthood [38]. Further, infants who were stunted at 1st month had 10.8 times higher odds of having low HC [28]. This was also true at the end of first and second year with stunted infants having 3.1 and 2.6 times higher odds of low HC respectively.

HC is an indicator of stunting or chronic malnutrition and our study has not only reflected the stunting proportions similar to the proportions estimated by the WHO for the South-east Asian region as well as by the National Family Health Suryey-4, but also elicited strong association with HC [3, 39]. Also, children who started early in life with a normal HC, showed lower HC-for-age, later by their first birthday. This is probably because HC is also influenced by other factors such as exclusive breast-feeding and complementary feeding practices that play a pivotal role in the first year of life, though our study did not elicit a significant difference with HC and exclusive breast feeding [16, 40]. Following the first year of life, HC probably remains unaltered as observed in our study. The low HC established by the end of first year continues to persist and probably co-exists with concurrent malnutrition in these children. This emphasizes that HC at birth predominantly determines HC later with the first year of life being a highly critical period for achieving optimal growth. Also, the first year of life is a golden period to intervene and help children catch-up growth. Hence HC measurement at birth, and further serial measurements up to the first year of life is a pragmatic and a highly informative parameter to monitor children who could potentially slip into the cascade of malnutrition, as this is the best period when interventions are plausible and effective.

Our study showed that socio-economic status had a significant association with a low HC and this is similar to the findings from a study in eastern India [4]. High prevalence of maternal undernutrition that sets in as early as adolescence in girls from impoverished communities could be a biologically plausible explanation for this [41]. Assuming a normal micronutrient status to start with at birth, we measured micronutrient status and inflammation at the 7th month. Anaemia was observed in about half the infants but did not show significant association with low HC. Underlying inflammation flagged by an elevated α-1-acid glycoprotein was seen in a third of the infants and this showed no significant differences between those with low and normal HC. Children who measured for normal HC at birth and later had not caught up with the expected HC could possibly be due to an enteropathy setting in early following birth as shown by a study on Zimbabwean children where enteropathy in the background of inflammation was associated with stunting [42]. However, our study did not elicit this. Low maternal IQ was found to be associated with low HC and this is compounded with findings from the same geographical area by Anoop et al. where low maternal intelligence was associated with malnutrition in infants [43]. Low paternal and maternal HC were the strongest associations with low HC in children in our study and this shows that apart from possible external exposures, it was the genetic influence that strongly determined HC in infants as put forth by Silventoinen et al. [44]. Overall, it can be said that parental characteristics encompassing parental nutritional status and their early exposure in-utero along with the living standards and economic conditions perhaps amalgamate directly or indirectly to influence the HC and thereby malnutrition in children. The unavailability of HC and length immediately following birth along with the gestational age that did not permit us to adjust our anthropometric measurements at all time-points, was a limitation for our study.

It can be concluded that HC measurements along with routine length/height and weight can play a pivotal role in predicting stunting as shown by the relationship between stunting and HC elicited by our study. Health systems in developing countries should thereby have a systematic approach to the recording of these simple yet vital measurements beginning from birth. Immunization visits provide a valuable opportunity to document HC along with weight and length measurements early in the first year of life. It is also simple tool where mothers can be taught to measure HC in their infants especially in difficult settings as demonstrated by studies where a high degree of agreement was elicited between the anthropometrist and parental measurements of HC [45]. Also, developed countries have established normative databases for head circumference of their populations that the developing countries lack [46]. Developing countries like India need to establish the same, as comparison and interpretation of its data with international charts may not be suitable to draw precise and valid conclusions for all ethnic settings [47, 48]. Establishing normative data on HC for Indian population could play a cardinal role in further understanding HC in the Indian setting and in the long, have policy implications on the timing and package of interventions to curb the problem of stunting.

## Conclusion

Children in the MAL-ED cohort established in the semi-urban settlement of Vellore started their life with a reduced head circumference, and the numbers further increased by the end of 2 years. Further, children who recorded low head circumference at birth continued to have low circumference at all time-points until 2 years of age. Stunting was significantly associated with low head circumference in the first 2 years of life, hence proving as an important tool of measurement apart from length/height and weight to predict stunting. Paternal and maternal head circumference were significantly associated with a reduced head circumference in children indicating a strong genetic influence. There is a definite need for the establishment of normative data for head circumference for both children as well as adults for the Indian population. Head circumference measurement, often not utilised optimally, can be a very simple tool that can be used by mothers and caregivers for growth monitoring at homes thereby help in early detection of growth faltering.

## Additional file

Additional file 1: **Table S7.** Comparison of baseline characteristics of the children who completed the two-year follow-up with those who were lost-to-follow up. (DOCX 19 kb)

## Data Availability

The datasets used and analysed during the current study are available from the corresponding author on reasonable request.

## Abbreviations

CI: Confidence Interval
HC: Head Circumference
HPLC: High Performance Liquid Chromatography
IQ: Intelligent Quotient
LCECU: Low Cost Effective Care Unit
MAL-ED: The Aetiology, Risk Factors, and Interactions of Enteric Infections and Malnutrition and the Consequences for Child Health study
OR: Odds-ratio
RCM: Raven’s Combined Progressive Matrices instrument
SD: Standard Deviation
UHC: Urban Health Centre
WAMI: Socio-economic status index that includes access to improved water and sanitation, eight selected assets, maternal education, and household income
WHO: World Health Organization
