# Supplementary material for: Low head circumference during early childhood and its predictors in a semi-urban settlement of Vellore, Southern India
Source: BMC Pediatr. 2019 Jun 6;19:182. doi: 10.1186/s12887-019-1553-0 (PMC6552319; doi:10.1186/s12887-019-1553-0)
Supplement: Supplementary file 1 — Table S7. Comparison of baseline characteristics of the children who completed the two-year follow-up with those who were lost-to-follow up. (DOCX 19 kb) [file 12887_2019_1553_MOESM1_ESM.docx]

Additional file 1: **Table S7** Comparison of baseline characteristics of the children who completed the two-year follow-up with those who were lost-to-follow up

|  | |  | ***Completed follow up (N=228)*** | | ***Lost-to-follow up (N=23)*** | | ***P-*value** |
| --- | --- | --- | --- | --- | --- | --- | --- |
| **Variable** | | **Category** | ***n*** | **%** | ***N*** | ***%*** |  |
| **Gender** | | Male | 105 | 46 | 8 | 35 | 0.3 |
|  |  | Female | 123 | 54 | 15 | 65 | - |
| **Birth weight (kg)** | | Very Low Birth weight (<1.99 kg) | 5 | 2 | 0 | 0 | 1.00* |
|  |  | Low birth weight (2 - 2.49 kg) | 32 | 14 | 3 | 14 | - |
|  |  | Normal birth weight (≥ 2.5 kg) | 186 | 84% | 19 | 86 | - |
| **Socio-economic status (WAMI^a^)** | **6^th^ month** | Low (≤ 33^rd^ centile) | 69 | 31% | 5 | 71 | 1.00* |
|  |  | Middle and High (> 33^rd^ centile) | 156 | 69% | 2 | 29 | - |
|  | **12^th^ month** | Low (≤ 33^rd^ centile) | 74 | 32% | 0 | 0 | - |
|  |  | Middle and High (> 33^rd^ centile) | 154 | 68% | 1 | 100 | - |
|  | **18^th^ month** | Low (≤ 33^rd^ centile) | 72 | 32% | 0 | 0 | - |
|  |  | Middle and High (> 33^rd^ centile) | 156 | 68% | 0 | 0 | - |
| **Parity of the mother** | | > 2 | 91 | 40% | 9 | 39 | 0.916 |
|  |  | ≤ 2 | 135 | 60% | 14 | 61 | - |
| **Age of the mother** | | < 23 years | 96 | 42% | 7 | 30 | 0.264 |
|  |  | ≥ 23 years | 130 | 58% | 16 | 70 | - |
| **Mother’s education** | | No schooling | 26 | 11% | 3 | 13 | 0.823 |
|  |  | Primary (1^st^ to 5^th^ grade) | 54 | 24% | 6 | 26 | - |
|  |  | Secondary (6^th^ to 10^th^ grade) | 110 | 49% | 9 | 39 | - |
|  |  | High school (>11^th^ grade) | 36 | 16% | 5 | 22 | - |
| **Father’s education** | | No schooling | 30 | 14% | 1 | 100 | - |
|  |  | Primary (1^st^ to 5^th^ grade) | 56 | 26% | 0 | 0 | - |
|  |  | Secondary (6^th^ to 10^th^ grade) | 109 | 52% | 0 | 0 | - |
|  |  | High school (>11^th^ grade) | 17 | 8% | 0 | 0 | - |
| **Mother’s BMI** | | Under-weight (< 18.5) | 46 | 20% | 3 | 20 | 1.00* |
|  |  | Normal (18.5 - 24.9) | 132 | 59% | 9 | 60 | - |
|  |  | Over weight (≥ 25) | 48 | 21% | 3 | 20 |  |
| **Father’s BMI** | | Under-weight (< 18.5) | 20 | 10% | 0 | 0 | - |
|  |  | Over weight (≥ 25) | 54 | 26% | 0 | 0 | - |
|  |  | Normal (18.5 - 24.9) | 131 | 64% | 1 | 100 |  |
| **Mother’s IQ** | | Low (≤ 33^rd^ centile) | 81 | 36% | 1 | 20 | 0.659 |
|  |  | Normal and High (> 33^rd^ centile) | 147 | 64% | 4 | 80 | - |

*Fisher exact test
